# Occupational profiling driven by online job advertisements: Taking the data analysis and processing engineering technicians as an example

**DOI:** 10.1371/journal.pone.0253308

**Published:** 2021-06-22

**Authors:** Lina Cao, Jian Zhang, Xinquan Ge, Jindong Chen

**Affiliations:** 1 School of Economics and Management, Beijing Information Science and Technology University, Beijing, China; 2 Laboratory of Big Data Decision Making for Green Development, Beijing, China; 3 Beijing International Science and Technology Cooperation Base of Intelligent Decision and Big Data Application, Beijing, China; Vellore Institute of Technology: VIT University, INDIA

## Abstract

The occupational profiling system driven by the traditional survey method has some shortcomings such as lag in updating, time consumption and laborious revision. It is necessary to refine and improve the traditional occupational portrait system through dynamic occupational information. Under the circumstances of big data, this paper showed the feasibility of vocational portraits driven by job advertisements with data analysis and processing engineering technicians (DAPET) as an example. First, according to the description of occupation in the Chinese Occupation Classification Grand Dictionary, a text similarity algorithm was used to preliminarily choose recruitment data with high similarity. Second, Convolutional Neural Networks for Sentence Classification (TextCNN) was used to further classify the preliminary corpus to obtain a precise occupational dataset. Third, the specialty and skill were taken as named entities that were automatically extracted by the named entity recognition technology. Finally, putting the extracted entities into the occupational dataset, the occupation characteristics of multiple dimensions were depicted to form a profile of the vocation.

## Introduction

A complete occupational information system is convenient for institutions and groups such as government agencies, social service institutions, higher education institutions, enterprises and labor forces to obtain information on occupations and occupational skills effectively and in a timely manner. The traditional way of various countries in the world is to standardize and unify the occupational structure of the whole society through occupational classification standards [1]. The standards and job descriptions such as occupational job content, skill requirements and qualification requirements together form a vocational portrait system [2]. The representative networks are *the Occupational Information Network* (O*NET) in the United States [3], *European Skills, Competences, Qualifications and Occupations* (ESCO) in the European Union and *National Occupational Classification* (NOC) in Canada, etc.

In China, the traditional occupational portrait system includes the *Chinese Occupation Classification Grand Dictionary* (COCGD), which covers 1,481 occupations in the whole society [4], 1,055 vocational skill standards and qualification requirements for some occupations. These standards are basically able to comprehensively and objectively describe the professions in China. However, compared with Europe and the United States, the Chinese occupational portrait system still has some limitations. One is the missing vocational skills or qualification requirements of some vocations. Another limitation is that updates lag so that it is difficult to reflect career changes over time. For example, it has been 16 years since the current Occupational Classification Standard was updated. Additionally, the revision process usually consumes considerable time, manpower and resources. For example, the current version was revised by 74 departments and organizations and nearly 10,000 experts and scholars. Therefore, it is necessary to explore new methods to provide more diverse and real-time information for various groups to keep pace with a fast-changing employment sector.

At the same time, online recruitment has become an important channel for recruiters to release information and for job seekers to find jobs [5]. The content of the recruitment posts usually contains specific descriptions of the job information, specialty requirements, educational requirements, experience requirements, skill requirements, etc [6]. By extracting the requirement information from the recruitment text and aggregating it into effective vocational information, it can be used as an effective supplement to the traditional occupational profiling.

In fact, the emergence of massive data provides scientific research with a new paradigm of data-intensive research [7, 8], that is, the information, knowledge and wisdom needed to directly search or mine from the data. In 2007, Turing Prize winner Jim Gray distinguished data-intensive science from computing science as the Fourth Paradigm, which he argued may be the only systematic approach to some of the most intractable global challenges we face [9].

In the study of occupational profiling, there are two key technical problems in how to mining the characteristics of occupations. One is how to find the most relevant data about a specified occupation from the massive recruitment information. However, the existing study was driven by job advertisement mainly focused on the position analysis with the position data obtained by keyword searching. Another is how to extract the key requirements such as skills and specialties from the recruitment information. Some studies adopted the dictionary-based, statistics-based, and mix-based method to extract information, the application of the methods had some limitations.

The contribution of the paper are as follows:

- This work is a preliminary exploratory work to show the feasibility of vocational profiling driven by job advertisement. The proposed concept of the occupational profile can theoretically be seen as a new and feasible research branch.
- The similarity calculation and classification algorithm TextCNN were applied to find the job advertisements related to the target occupation.
- For methods of Chinese text extraction, the experiment compared the mainstream approach of named entity recognition and adopted the better model to predict new entities. This is a new technical attempt that expanded the application field of Chinese named entity recognition technology.
- The occupation characteristics of multiple dimensions were depicted.

The next section discusses the current state of research and various papers regarding the extraction of skills from job ads. Section 3 introduces our approach to occupational profiling and Section 4 describes the processing steps and details of each step. Finally, Section 5 gives the result analyses and Section 6 concludes the results and discusses future work.

## Related work

There were many researches on online recruitment data [10]. However, most of them took the recruitment position as the research object, and carries out the skills analyses based on the posts. Few researchers focused on the analyses of vocations based the online recruitment data. The difference between position and occupation should be pointed out. Generally speaking, one occupation includes a variety of positions. That is to say, a vocation is a collection of jobs that have the same identity.

### A. Occupational analysis

According to the existing literature, only the United States and the European Union had carried out the project of using online recruitment data to mine vocational skills information. The work report [11] of Australia’s Career Development Center pointed out that the acquisition and update of vocational skills information on O*NET in the United States, in addition to via the regular surveys of the skills, abilities and knowledge of the incumbents by occupation, area of tools and technologies, such as in the IT industry, were updated using online vacancy data as an effective supplement. However, there is currently no research literature describing the specific implementation details of O*NET in the United States.

In 2018, the European Center for Vocational Training and Development (Cedefop) released the Skills Online Vacancy Analysis Tool for Europe (Skills-OVATE). The project collected 30 million job advertisements in 18 EU countries and then used text classification technology to obtain occupational data, taking the European Occupational Classification Standard (ESCO) as the categorization standard. On this basis, it extracted key information such as skill words and provided eight functions including online recruitment occupation distribution statistics, popular occupations and skills analyses and industry and occupation relationship analyses. The project has shown great potential in promoting the formation of a more integrated and efficient labor market throughout Europe [12].

Two of the core technologies of the Skills-OVATE project were implemented through cooperation with scholars from the University of Milan Bicocca in Italy [13, 14]. In terms of text classification technology, Boselli et al. [13] used ESCO as the category label, extracted features through n-gram, and compared the supervised learning algorithms such as support vector machine (SVM) [15], random forest [16], and neural network. In terms of skill extraction technology, Boselli et al. [14] obtained candidate skill words through n-gram, and calculated their similarity with the string similarity of the skill label in ESCO. Those below the threshold were directly abandoned, and those above the threshold were judged by domain experts as existing skills, new skills or nonskills until the skills dictionary was stable. This method for extracting skills words requires the participation of domain experts, which is inefficient and costly. Additionally, since there are no skill labels such as ESCO in China that can be used as a selection standard, it is impossible to calculate the similarity value between skill words. Therefore, we tried computer methods to extract skill words more efficiently.

### B. Position analysis

In position research, the skill analysis was the most common of all. Thus, how to process the recruitment text is often the main core problem. Early text processing mainly relied on manual processing [17]. As the number of recruitment texts continued to expand, the use of computer technology had became a new trend.

The information extraction technological development process can be divided into three kinds: dictionary-based, statistics-based, and mix-based. 1) The dictionary-based method was the earlier and more adopted method. For example, Aken & Litecky [18] and Pejic-Bach et al. [19] constructed a skill dictionary or list and matched the dictionary with the recruitment text information to recognize skill keywords. The construction of the skill dictionary in this kind of method relies on expert experience; thus, the dictionary has low portability. 2) The method based on statistics mainly used the statistical attributes of the distribution of skill words to identify keywords. One type was to sort keywords by feature quantitative indicators; for example, Grüger & Schneider [20] used TF-IDF (term frequency–inverse document frequency) values to filter skill keywords. Statistical-based methods do not require syntactic and semantic information and do not rely on labeling data, but the accuracy rate is relatively low. Another type was based on probabilistic topic models to generate skills-related topics. For example, Gurcan & Cagiltay [21] used the Latent Dirichlet Allocation (LDA) model to obtain 48 potential topic-skill distributions, and Xu et al. [22] developed a novel skill popularity-based topic model (SPTM) for modeling the generation of the skill network. Skill words under each topic were ranked according to their popularity. However, topic models have problems such as difficulty in determining the number of topics and poor interpretability of model results. 3) Based on the hybrid method of rules and statistics, the effective integration of the two methods can effectively improve the performance of keyword extraction. For example, the SKILL system developed by CareerBuilder uses a word vector model to realize skill entity recognition and entity specification [23]. However, these artificial rules combined with traditional machine learning methods still need to be further improved in terms of versatility, scalability and machine learning performance.

With the development of deep learning technology, Jia et al. [24] and Wang Dongbo et al. [25] formalized the extraction of skill words into a type of named entity recognition (NER) task. The former was based on the long short-term memory (LSTM) algorithm to extract skill entities, and the latter was based on the conditional random field (CRF), LSTM-CRF and BiLSTM-CRF to extract entity related to data science positions. Among them, LSTM-CRF and BiLSTM-CRF, which were combined with deep learning, had better entity recognition performance than CRF due to the consideration of contextual information characteristics. Although the studies in the literature [24, 25] performed well on closed data sets, the generalization ability of the model for a large number of unknown words still needs further study. The bidirectional encoder representations from transformers (BERT) language preprocessing model proposed by Devlin et al. [26] further increased the generalization ability of the word vector model, which could fully describe character-level, word-level, sentence-level, and even inter-sentence relationship features [27–29]. However, there has been no attempt to apply BERT to named entity recognition in online recruitment data.

## Methods

### A. COCGD description

In COCGD (2015 Edition), jobs were divided into 8 major categories, 75 medium categories, 434 minor categories and 1,481 occupations. The hierarchical classification system reflects China’s economic structure, population and employment structure to a certain extent, and its description of the job tasks of each occupation was highly concise.

To verify the feasibility of using online recruitment data to carry out occupational profiling, the paper selected an occupation as an example to show the specific procedure. The selected vocation case was data analysis and processing engineering technicians (DAPET) which is subordinate to the minor categories management (industrial) engineering and technical personnel in COCGD. Its code is GBM2–02-30-09 and the description is “*engineering and technical personnel engaged in information system data planning, collection, management, analysis, database design and optimization, data resource integration, data mining, data analysis, etc. Their main tasks: analyze system data sources and data application requirements, design data resource integration solution; design the logic of the database; Implement database and data service application programming; adjust and optimize the database system; carry on the data analysis, data mining, data presentation and decision support; run and maintain database system; perform data and information processing, and provide data consulting services*.” After an inquiry, there is no vocational skill standard of the DAPET at present in China. Thus, it is necessary to mine the skill information of this vocation.

### B. Data description

The website 51job.com is one of the largest online recruitment platforms in China, and its job advertisements are divided into 11 categories and 65 categories according to the type of industry. However, this industry classification is different from the current industry classification standards of the national economy and different from the abovementioned COCGD. Therefore, the study needs to extract the required occupational data according to the description of occupations in COCGD.

A piece of recruitment data usually contains two parts: structured data and unstructured textual data. Structured data include the job title, company type, working city, salary and benefits, and education and experience requirements. The unstructured text data include the job responsibilities and job requirements. The former describes what the employee needs to do, whereas the latter requires what abilities the employee needs to have. According to the characteristics of the content of the online recruitment text, in this study, the job responsibility section was compared with the occupational description text in COCGD to screen and obtain the target occupation data; the job requirement section was mainly used to extract and analyze the skill and specialty requirements through the information extraction technology.

### C. Models and process

The specific realization process of occupational portraits involves the comprehensive and integrated use of multiple automatic text processing technologies.

**Fig 1:**
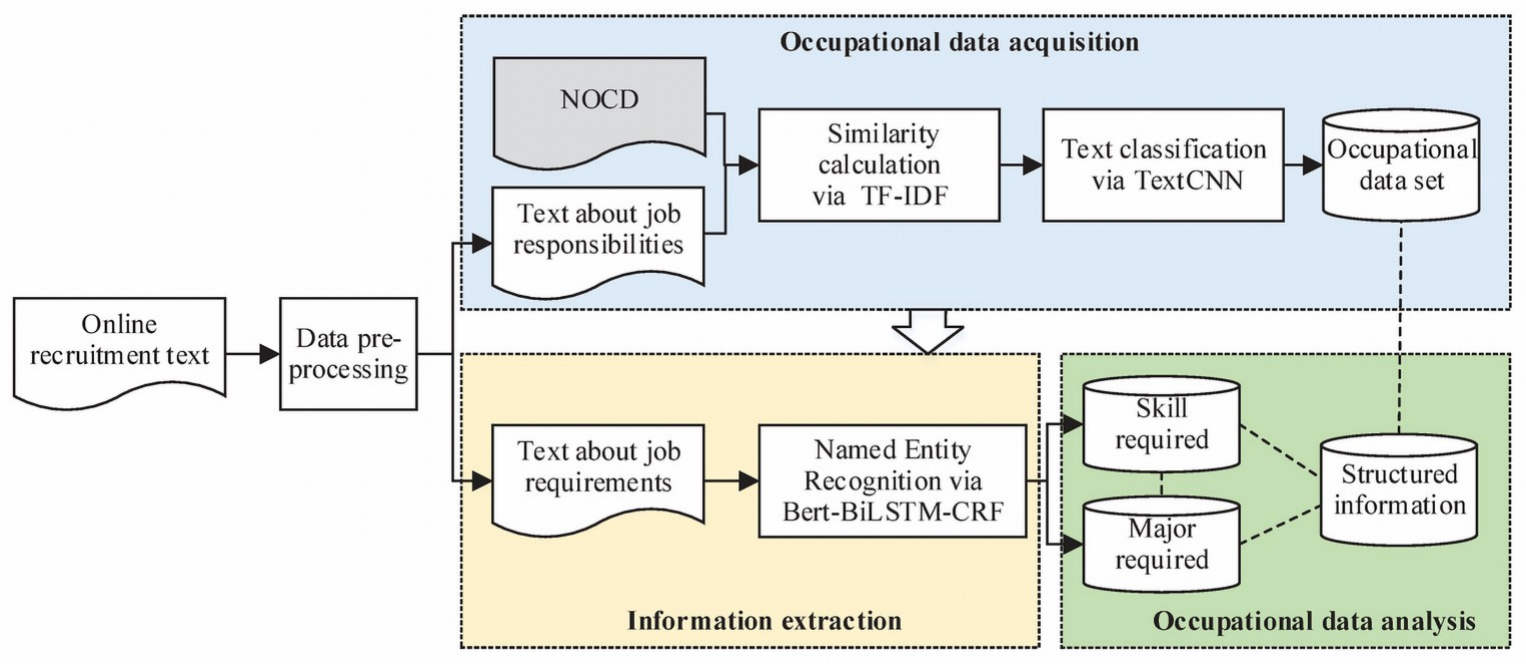
Flow chart of occupational portrait.

Fig 1 shows the specific methods and process. First, the source data were crawled and preprocessed. Second, the section of the text that describes the job responsibilities was used to calculate the similarity value with the National Occupational Classification Dictionary text through the TF-IDF vector, and a preliminary occupational dataset was obtained. Then, the initially screened data were further classified via the TextCNN model to obtain an accurate occupational dataset. Third, the BERT-BiLSTM-CRF model was used to carry out the named entity recognition task of this dataset to extract skill and specialty requirement information. Finally, according to the extracted information and other characteristics of other dimensions presented by the occupational dataset, such as salary and work location, the occupational characteristics were analyzed and described.

#### TextCNN model

After preliminary screening data by setting the similarity threshold, it is necessary to further use the classification model to accurately classify online recruitment data according to the occupational descriptions of DAPET.

Convolutional Neural Network (CNN) was originally built for image processing. Its structure is similar to the visual cortex, but it is also effectively used for text classification. Kim [30] first proposed the sentence classification model with a single convolutional layer structure of CNN to classify the text data. Different from the deep CNN used in image recognition, Convolutional Neural Networks for Sentence Classification (TextCNN) usually has only one convolutional layer and one pooling layer. This is because the text data dimension is far less than the image data dimension, TextCNN which contains only one layer of convolution and pooling can also have a better prediction result of classification, and multiple convolutional and pooling layers can cause overfitting instead.

**Fig 2:**
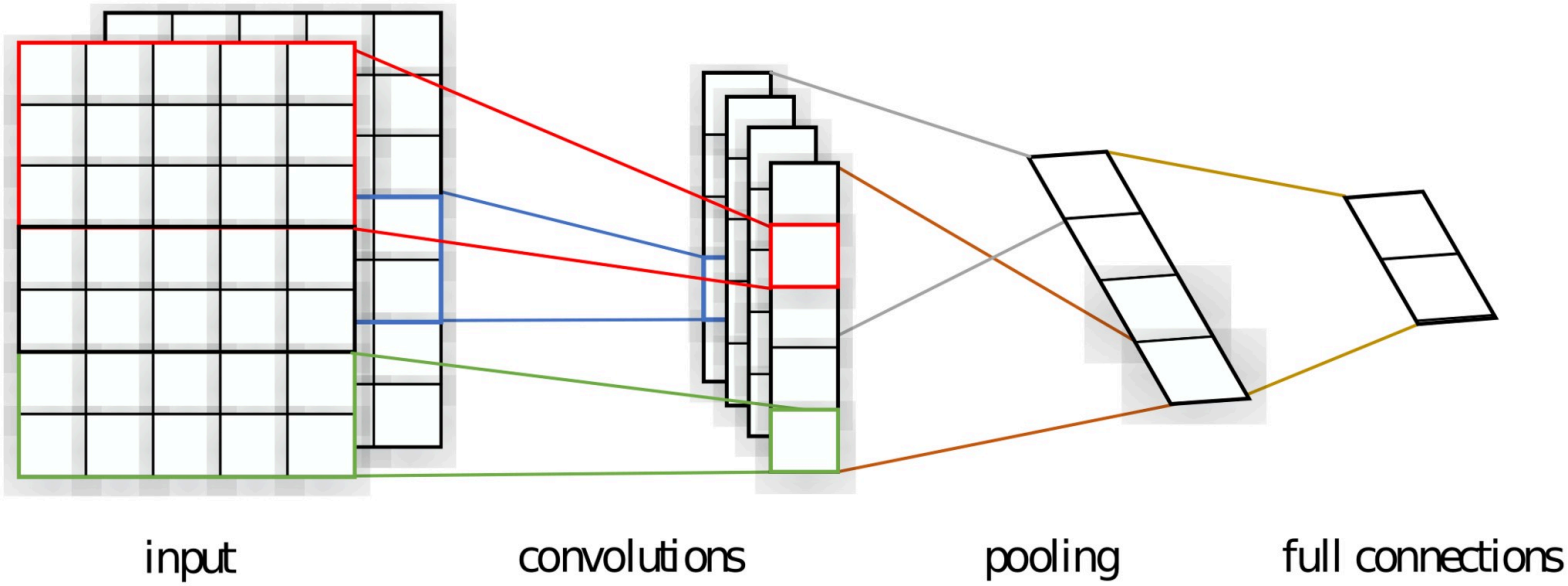
Framework of TextCNN model.

Compared with traditional machine learning methods, TextCNN model provides a direct end-to-end solution for text classification, which can avoid complex manual feature engineering. Fig 2 shows the structure of TextCNN model with two channels including input layer, convolution layer, pooling layer, full connection layer and output layer.

The input of TextCNN is a sequence of word vectors. There are three ways to initialize word vectors. The first one is random initialization; the second is static word vectors trained by pre-training models such as Word2vec [31], GloVe (Global Vectors for Word Representation) and FastText; the third is using dynamic word vectors such as Bert and XLNet. There are also two ways to train the word vector sequence of TextCNN. One is that the word vector is fixed in the whole process of BP (BackPropagation) algorithm after initialization, and the other is that the word vector sequence is updated with back propagation. Compared with the two, generally the latter can improve the classification effect.

The role of convolution layer is to extract text features through convolution operations. Different from the image task in which the convolution check is the sliding convolution process from left to right and top to bottom of the image, in TextCNN, the column dimension of the convolution kernel is the same as the dimension of the input matrix, and only slides on the columns of the matrix with a slide step length of 2–5 words.

After the feature mapping is completed in the convolution layer, the eigenvalues enter the pooling layer for pooling. The pooling function of TextCNN is the maximum pooling function, which aims to capture the most prominent eigenvalues. After that, the word vectors are connected to form the last hidden layer of the network, and then the corresponding classification output of the final layer is obtained through Softmax’s mapping operation from high dimension to low dimension vectors.

### BERT-BiLSTM-CRF model

In order to analyze the vocational characteristics, the requirements words of skills and specialties should obtain from the job requirements texts. However, as a specific type of named entity, entities such as skills and specialties are more difficult to extract than common types of named entity recognition. Therefore, this paper proposed to construct BiLSTM-CRF based on BERT word embedding vector to extract various terms, and compared with the BiLSTM-CRF model which was as a benchmark model [32].

The main difference between the BERT-BiLSTM-CRF model and other deep learning-based named entity recognition models is the addition of BERT pre-training Chinese vectors. The BERT pre-training vector was learned by Google on a large-scale Chinese corpus. The vector representation of characters can be calculated through the text context, which can characterize the ambiguity of characters and enhance the semantic representation of sentences.

**Fig 3:**
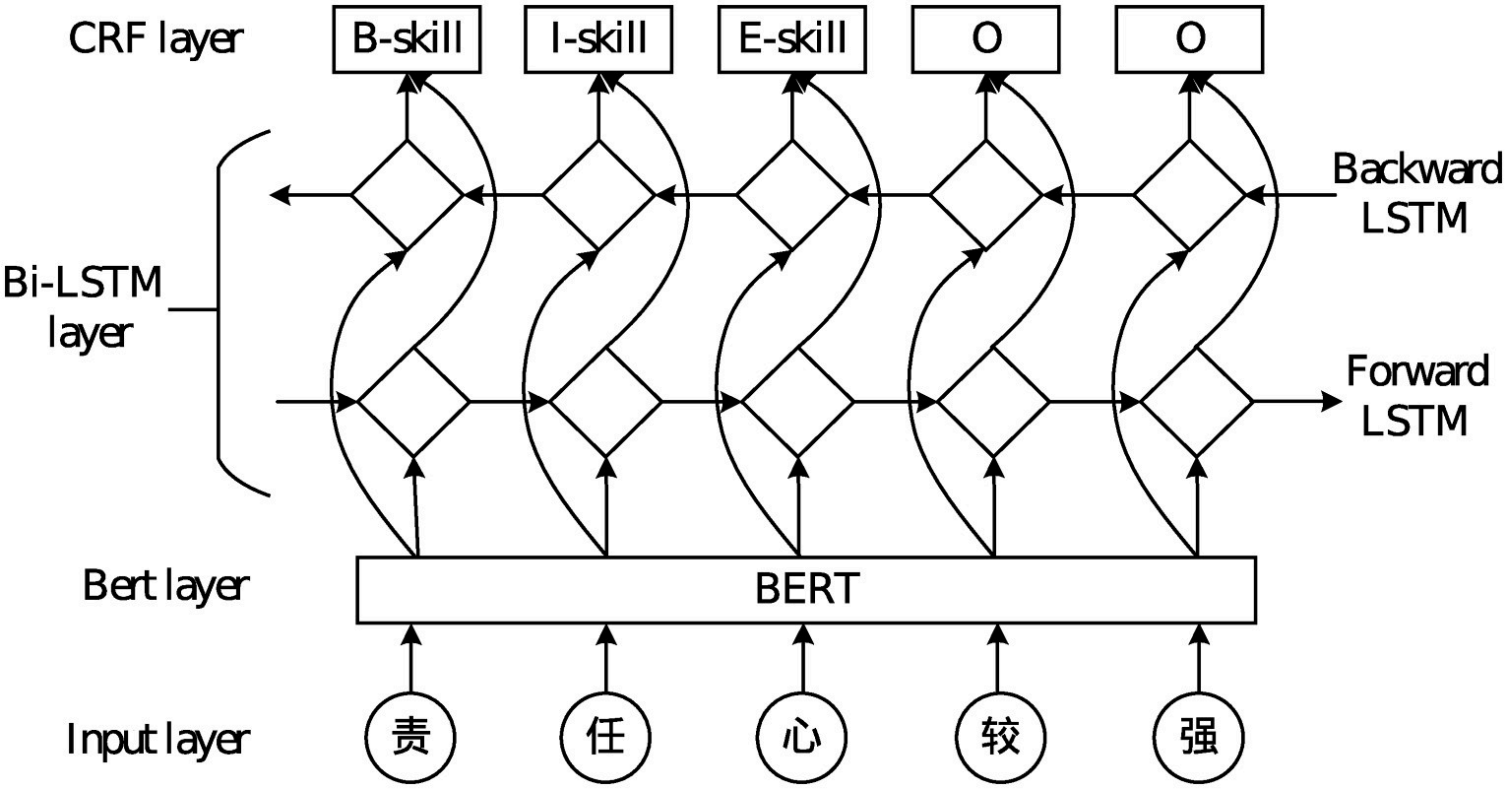
Framework of BERT-BiLSTM-CRF model.

The BERT-BiLSTM-CRF model can be divided into 3 parts as shown in Fig 3 below. The BERT layer obtains the semantic representation of the input corpus characters through the pre-training vector, and after obtaining the vector representation of each word in the sentence, the word vector sequence is input into the BiLSTM deep neural network for further semantic encoding. In the BiLSTM layer, a forward LSTM computes a representation of the sequence from left to right, which is designed to learn word prefix information; another backward LSTM computes a representation of the same sequence in reverse, which is aimed to learn suffix information [33]. This layer combines the front-to-back information and the back-to-forward information of the data for learning, thereby improving the semantic interaction between words [34]. Finally, the CRF layer is used to decode the output of the BILSTM module to get a prediction labeled sequence, and then each entity in the sequence is extracted and classified, so as to complete the whole process of Chinese entity recognition.

## Experiment

### A. Data obtaining and preprocessing

This paper used a web crawler developed by Python to capture all the job advertisements of 51job.com from June 6th to June 23rd, 2020, with a total number of 3,655,953. Each piece of data included nearly 20 dimensions such as recruitment position name, educational requirement, years of experience requirement, job responsibility requirement, employment requirement, recruitment enterprise type, and enterprise size.

In the data preprocessing stage, the raw data were deduplicated and denoised. Then, the recruitment texts were divided into two sections: job responsibilities and job requirements. Each section was cut into words and the stop words were removed. After that, data with fewer than 5 words in either were removed. The number of processed data was 2,884,625.

### B. Preliminary corpus construction

To obtain the data related to DAPET occupation from a large number of job advertisements, the research used the method of calculating the similarity value first for preliminary screening and then classification instead of direct classification. This avoided the problems of sample imbalance and excessive resource consumption in the classification task under large-scale data. First, the cosine similarity score between the description text of DAPET and responsibility text of each job were computed by using TF-IDF in feature vectors and obtain the sort of similarities. Then, the screening threshold was set to 0.25 by artificial judgment ensuring that relevant occupational texts were all included. Finally, 15,172 occupational texts of DAPET were screened out.

### C. TextCNN model further determines the occupational data set

Due to the cautious setting of the threshold, it is still necessary to further confirm the occupational dataset through the classification model. To this end, 3,000 pieces of data were randomly selected from 15,172 pieces of data for manual labeling, that is, according to the requirements and description of job responsibilities, compared with occupation descriptions of DAPET to artificially judge whether they belong to DAPET occupation. This labeling is dichotomous. After obtaining the labeled sample set, the TextCNN model was used for training. The model was implemented using the open-source toolkit “textclf” on github.com. During the training process, word vector initialization was selected randomly and continuously updated, an adaptive momentum estimation optimization (Adam) method was utilized and the adaptive adjustment method was used to adapt the parameter learning rate.

The training adopted the method of 10-fold cross validation and measured the model effect through three indexes: accuracy, recall rate and F value. The average results of ten experiments were 84.31% accuracy rate, 86.62% recall rate, and 84.88% F value. After batch processing with this model, an accurate dataset with 5,869 pieces of data highly related to DAPET occupation was obtained.

### D. BERT-BiLSTM-CRF model extracted the named entities

Entities of skill requirements and specialty requirements were taken as named entities of specific types, and the deep learning model was used to automatically extract them. Thus, the BiLSTM-CRF model and the BiLSTM-CRF model based on the Bert word embedding vector were constructed. Then, the two models were compared, and the model with higher accuracy was selected to realize the automatic extraction of named entities in large quantities from occupational data.

#### Data annotations

We used the BIOES scheme for text annotation, where B represents the beginning of an entity, I represents the inside, E represents the end, S represents a single entity and O represents the out. More than 5,000 pieces of data were randomly selected from all data for manual annotation and more than 60,000 entities were labeled in the experiment. During the annotation task, a tool named Colabeler was used to improve efficiency.

#### Experimental environment

All experiments were performed on the Ubuntu 16.04 system with four Inter Core CPU i7–6700 processors(3.40GHz), 16 GB of memory and an NVIDIA GeForce GTX 1060 GPU.

#### Parameter setting

To ensure the rigor of the experimental results, we conducted experiments through two deep learning algorithms on the same corpus after the same processing and under the same experimental environment. After exploratory experiments in the early stage, this study determined the optimal setting of hyperparameter settings which are shown in Table 1.

**Table 1:** Model parameter setting.

Model	Parameter setting
BiLSMT-CRF	Hidden unit is 256, batch size is 64, dropout is 0.5, learning rate is 0.001, epoch is 100 and clip is 5.
Bert-BiLSTM-CRF	Max sequence length is 256, train/dev/test batch size is 32, 8, 8, learning rate is 5E-5, epoch is 10, dropout is 0.5, clip is 5, and hidden unit is 256.

#### Experimental process and result

The 5000 sample data were divided into a training dataset, validation dataset and test dataset at a ratio of 8:1:1. The performance of the model was tested by the method of 10-fold cross-validation, and the effectiveness of the named entity recognition of the model was assessed using the precision rate, recall rate and F-score. The calculation method of each index is as follows, where A, B and C represent the number of correctly identified, wrongly identified and unidentified entities respectively.

$$\begin{array}{cc} \text{Precision}\;\text{rate}:\;P=\frac{A}{A+B} & \times 100\% \end{array}$$ (1)

$$\begin{array}{cc} \text{Recall}\;\text{rate}:\;R=\frac{A}{A+C} & \times 100\% \end{array}$$ (2)

$$\begin{array}{cc} F\;\text{value}:\;F=\frac{2\times P\times R}{P+R} & \times 100\% \end{array}$$ (3)

In validation and testing, an entity was considered to be correctly labeled only if its type and start-stop boundary were both identified correctly. The mean values of ten experimental results of the two models are shown in Table 2.

**Table 2:** Comparison of entity recognition results of models.

Named entities	Evaluation index	BiLSTM-CRF model			Bert-BiLSTM-CRF model		
		Precision Rate (%)	Recall Rate (%)	F-score (%)	Precision Rate (%)	Recall Rate (%)	F-score (%)
Specialty entity		92.36	90.56	91.45	95.98	93.55	94.75
Skill entity		83.25	84.43	83.84	85.88	87.45	86.66

Table 2 shows that the recognition effect of the two models on specialty entities was better than that on skill entities. This is because the sentence expressions of major entities are relatively single and easy to identify. Meanwhile, compared with the BiLSTM-CRF model, the recognition effect of the model based on the Bert pretraining vector was better. In the latter, the average F-value of major entity and skill entity recognition were 94.75% and 86.66%, respectively, which were slightly higher than those of the former of the benchmark model BiLSTM-CRF of 91.45% and 83.84%. This showed that the use of the Bert Chinese pretraining vector could effectively improve the performance of the whole model and had certain reference significance for other similar serialized entity recognition tasks.

Finally, the trained automatic named entity extraction model was used to predict the named entity of 5,869 occupational data. Then, the specialty entities and skill entities in each job requirement text were obtained.

## Result analysis

To describe the occupational characteristics of data analysis and processing engineering technicians, the paper conducted individual and overlapping analyses from the attributes of the position category, degree requirement and experience requirement together with the extraction results of skill and specialty entities.

### A. Overall analysis

#### What positions does the occupation include?

**Fig 4:**
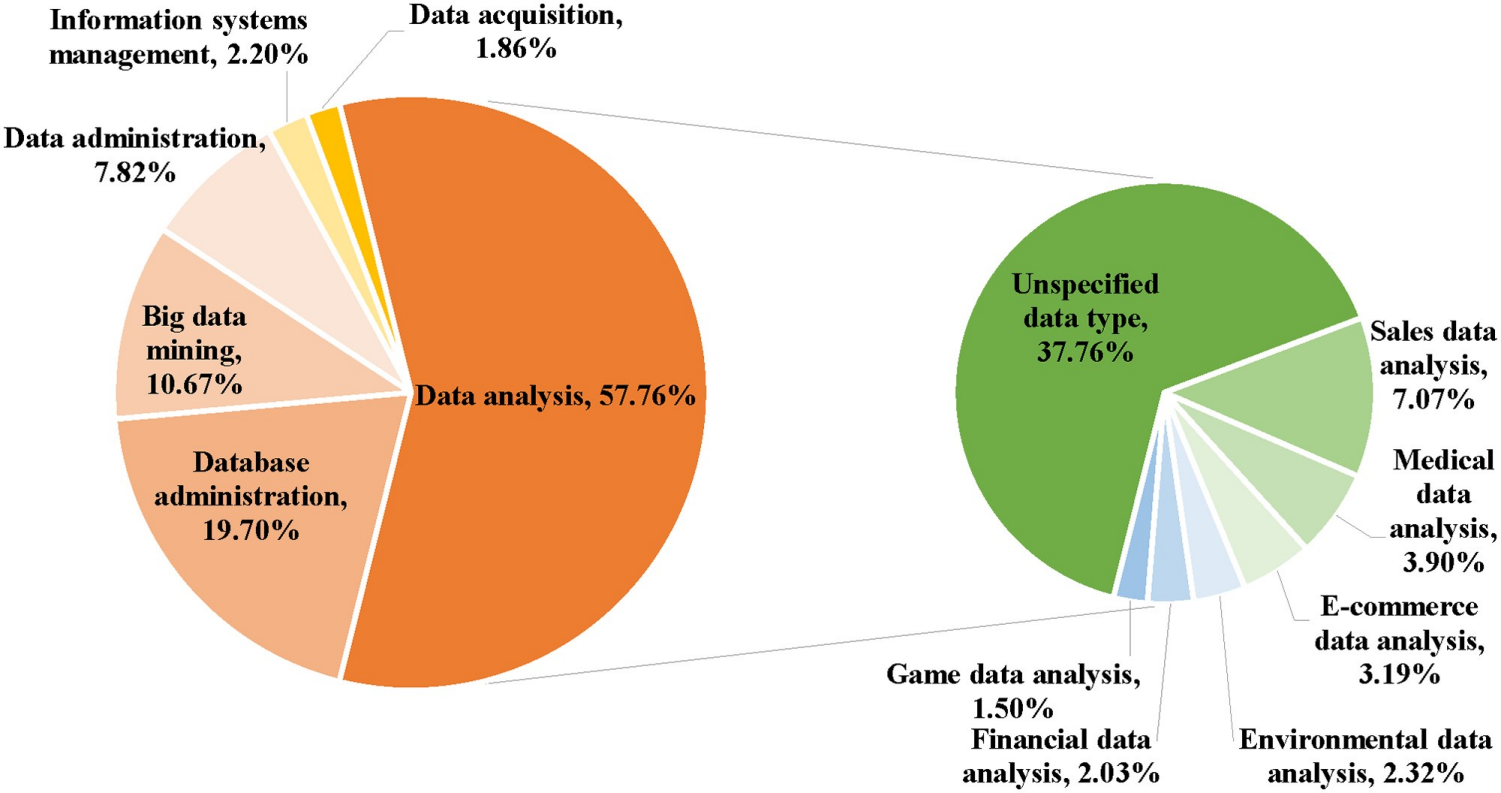
Categories of positions and their proportions.

Using the K-means cluster analysis method combined with manual work, the occupational dataset can be summarized into six clusters mainly according to the position names. Their names and proportions can be seen in Fig 4: the data analysis position has the highest proportion, accounting for 58% of all positions; the second and third are positions of database administration and big data mining, accounting for 20% and 11%, respectively; the remaining positions are data administration, information systems management and data acquisition, accounting for 12% total; among the data analysis jobs, except for 37% that are of an unspecified data type, the remaining 20% of the positions can be subdivided into seven categories according to the data type or industry of data applications such as sales data, medical data and e-commerce data.

#### What skills does the occupation require?

**Fig 5:**
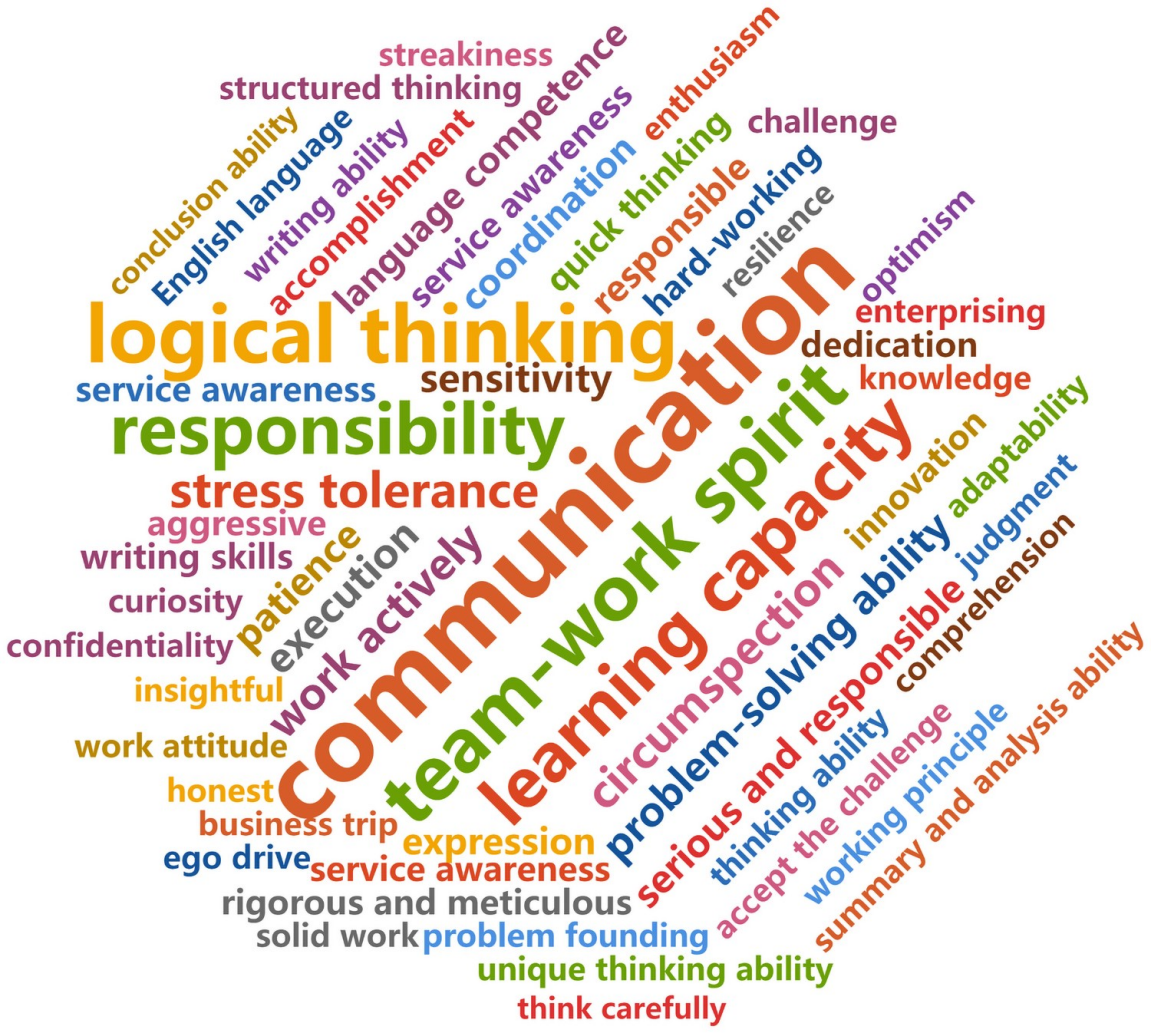
Cloud map of core skill words.

**Fig 6:**
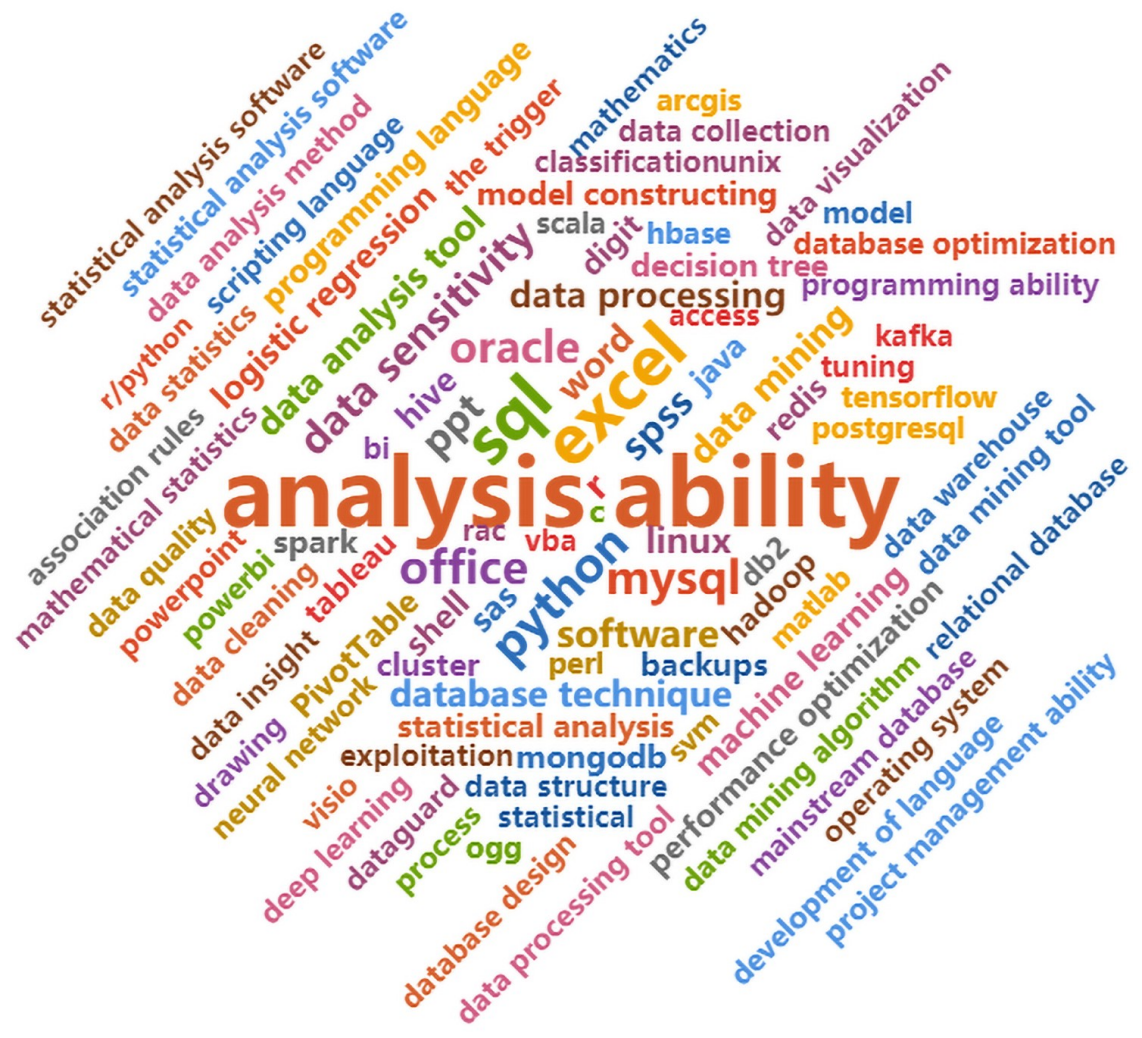
Cloud map of professional skill words.

The statistical results of the extraction of skill words are shown as the cloud charts in Figs 5 and 6. Fig 5 shows the top words of core skills (general skills) required by DAPET. The abilities mentioned focused on communication ability, sense of responsibility, logical thinking, teamwork spirit, responsibility and other soft skills [35]. Fig 6 shows the top professional skills required by the occupation, including data analysis ability, data sensitivity and data analysis tools such as Excel, SQL, Python and SPSS. Therefore, from the perspective of the labor supply side, the general skills and professional skills of workers are equally important.

According to the statistical results of skill words, data analysis and processing engineering technicians usually require comprehensive skills. First, a certain business sensitivity is required to perceive the industry or business and to understand the data in the business. Second, tools that can extract data are needed, including SQL, MySQL, and the models and tools for analyzing data, such as Python, R and SPSS. Third, data sensitivity is needed to interpret the data. Finally, tools such as PPT are needed to display data.

#### What specialties does the occupation require?

**Fig 7:**
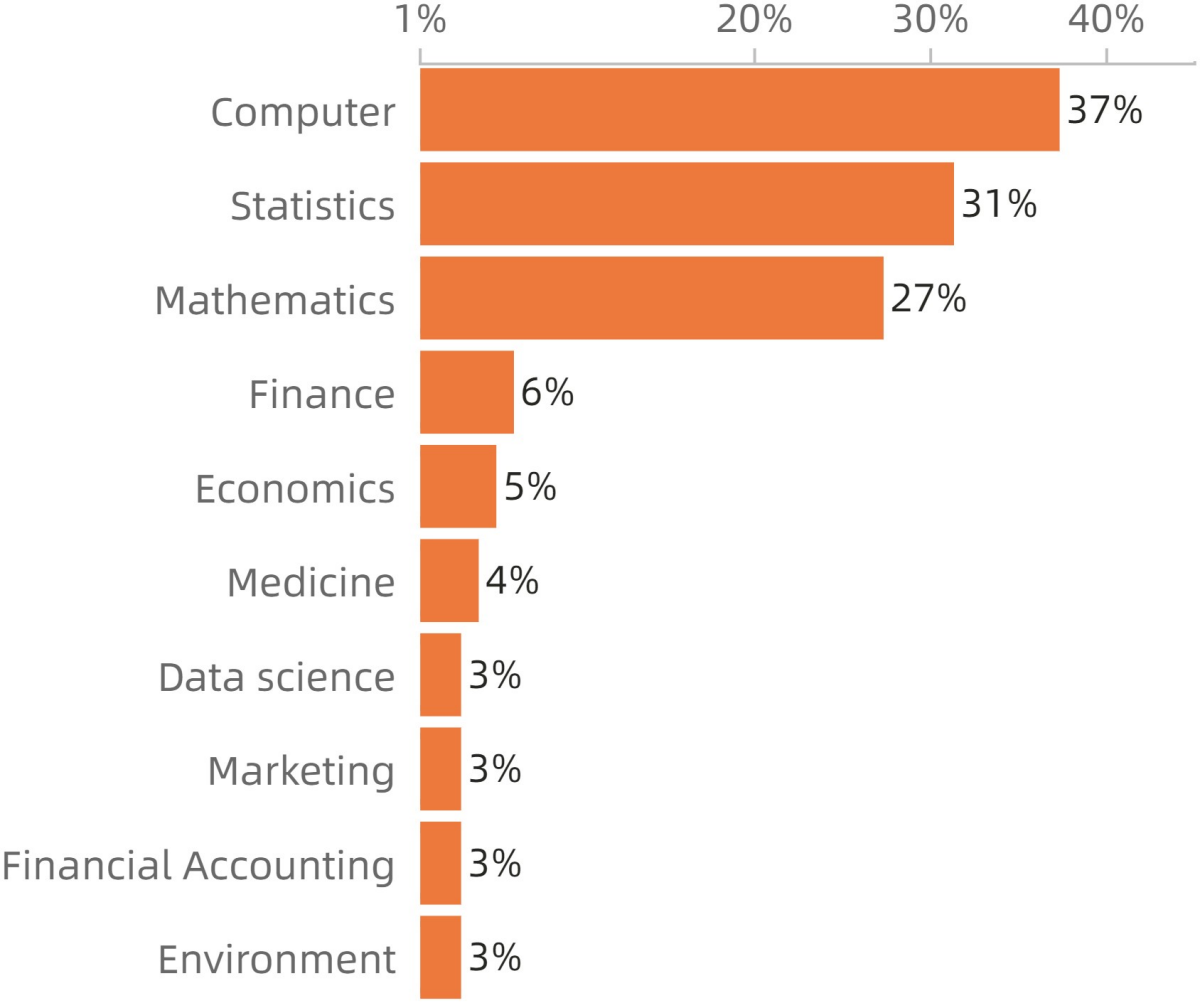
Order of ratio of professions requirement in DAPET.

According to the BER-BiLSTM-CFR model results and statistics, 3614 pieces of online job postings were extracted for specific specialties. Counting the frequency of required specialty entities and then calculating and sorting the proportion of each major in all frequencies of the mentioned majors in occupational data, the popular majors required by DAPET occupation were obtained, as shown in Fig 7. This indicates that the most desirable majors for this vocation are computer, statistics and mathematics, which are mentioned in 37%, 31% and 27% of the 5,869 occupational data samples, respectively. There are also other majors such as finance, economics, medicine, marketing, financial accounting and environment, which are required when considering the application scenarios or industries of data analysis and processing. Unexpectedly, the majority of medicine and environment rank sixth and tenth, respectively, due to the emerging demands of medical and environmental data analysis.

Note that the majority of data science ranked seventh among all majors, with 175 mentions in the job advertisement of data analysis. However, in China, the major named data science and big data technology is actually the most appropriate major for this occupation. The specialty was set up in colleges and universities for 2016 undergraduate programs with four years of schooling, aiming to cultivate interdisciplinary talents with big data processing and analysis ability. In 2020, the first batch of students of this major graduated. Obviously, according to the data result, the social awareness of this major is not high. However, to meet the needs of society, the teaching objective of this major should be to cultivate talent with comprehensive skills including mathematical statistics, computer technology and basic mathematics.

### B. Overlapping analysis

#### What’s the difference between those positions?

To analyze the difference of those positions, the professional skill words were measured and ordered according to the position categories. Then, the skill words were divided into two kinds: cognitive ability and practical ability. The former refers to the concepts, principles and methods related to the field, while the latter refers to a tool or programming language that translates knowledge into practice. Table 3 shows the top 5 knowledge skill words and the top 10 tools or programming language respectively in each position.

**Table 3:** Order of professional skill words in each position.

Requirement	Skill	Position	data analysis	Big data mining	Data administration	Data acquisition	Database administration	Information systems management
Cognitive ability			analysis ability	analysis ability	analysis ability	ModBus	performance optimization	information security
			data processing	data mining	data management	OPC	backups	network technique
			data mining	machine learning	data statistics	HTTP	data storage	system administration
			logistic regression	logistic regression	data quality	PROFINET	troubleshooting	cyber security
			machine learning	programming language	data processing	TCP/IP	database configuration	medical informatization
Practical ability			excel	Python	excel	PLC	SQL	SQL
			SQL	SQL	office	Java	Oracle	Office
			office	R	PPT	Python	MySQL	Oracle
			PPT	hive	SQL	C/C++	Linux	GIS
			Python	excel	Python	HTML	Shell	CAD
			SPSS	SPSS	R	word	MongoDB	ERP
			R	SPARK	MySQL	excel	Python	Windows OS
			word	Hadoop	word	C#	Redis	ArcGIS
			SAS	Java	SAS	MySQL	Perl	.NET
			PivotTable	SAS	PivotTable	Hadoop	PostgreSQL	ISO standards

The positions of data analysis and data mining are quit similar: they both required data analysis ability, data mining ability and knowledge of machine learning and logistic regression. The difference is that they give different weights to the most frequently used method of statistical analysis software, programming language and database management software. In addition, high-performance computing technologies such as data warehouse platforms and cluster computing environments are key skills when engaging in massive data storage, computing and mining. The position of data administration requires the abilities of data processing, statistics, analysis and management, and also the knowledge of how to improve the data quality. The tools required for this position are similar in difficulty to those needed for data analysis. The position of data acquisition requires familiarity with common industrial communication protocols, such as Modbus and Profinet, network communication protocols, such as HTTP and TCP/IP, and data transmission specifications such as OPC, and requires the common programming languages such as Java, Python and C, programmable logic controller (PLC) and HyperText Markup Language (HTML). The position of database administration requires mastering the knowledge of database management which contains performance optimization, data storage and backups, troubleshooting, configuration and so on, and mastering the mainstream database management software, operation system and data memory system such as redis, without much emphasis on programming ability. Information systems management requires knowledge of information security, network techniques, system administration, cybersecurity, and informatization knowledge for specific industries. The tools of this position required to master include database software and information systems for various industries include geographic information system (GIS), enterprise resource planning (ERP) system.

Additionally, the required number of skills in each position is accounted in Table 4. Approximately two professional skills are required on average in each job advertisement. The professional skills included knowledge skills and tools, excluding core skills. Combining Tables 2 and 3, shows the varying degree of difficulty in engaging in different jobs. The big data mining position has the highest requirement regardless of the number of skills or the terms of professional skills. Jobs of data analysis and database administration need roughly the same number of skills, but the latter emphasizes database capabilities. Jobs of data administration, information systems management and data acquisition require fewer skills but comprehensive abilities.

**Table 4:** Statistics of skills in each position.

Positions	Number of job advertisement in each position	Total frequency of professional skills required	Average number of professional skills requires in each job advertisement
Big data mining	626	1750	2.8
Data analysis	3390	6909	2.0
Database administration	1156	2078	1.8
Data administration	459	709	1.5
Information systems management	129	149	1.2
Data acquisition	109	128	1.2
Total	5869	11723	2.0

#### What is the educational requirement of the occupation?

**Fig 8:**
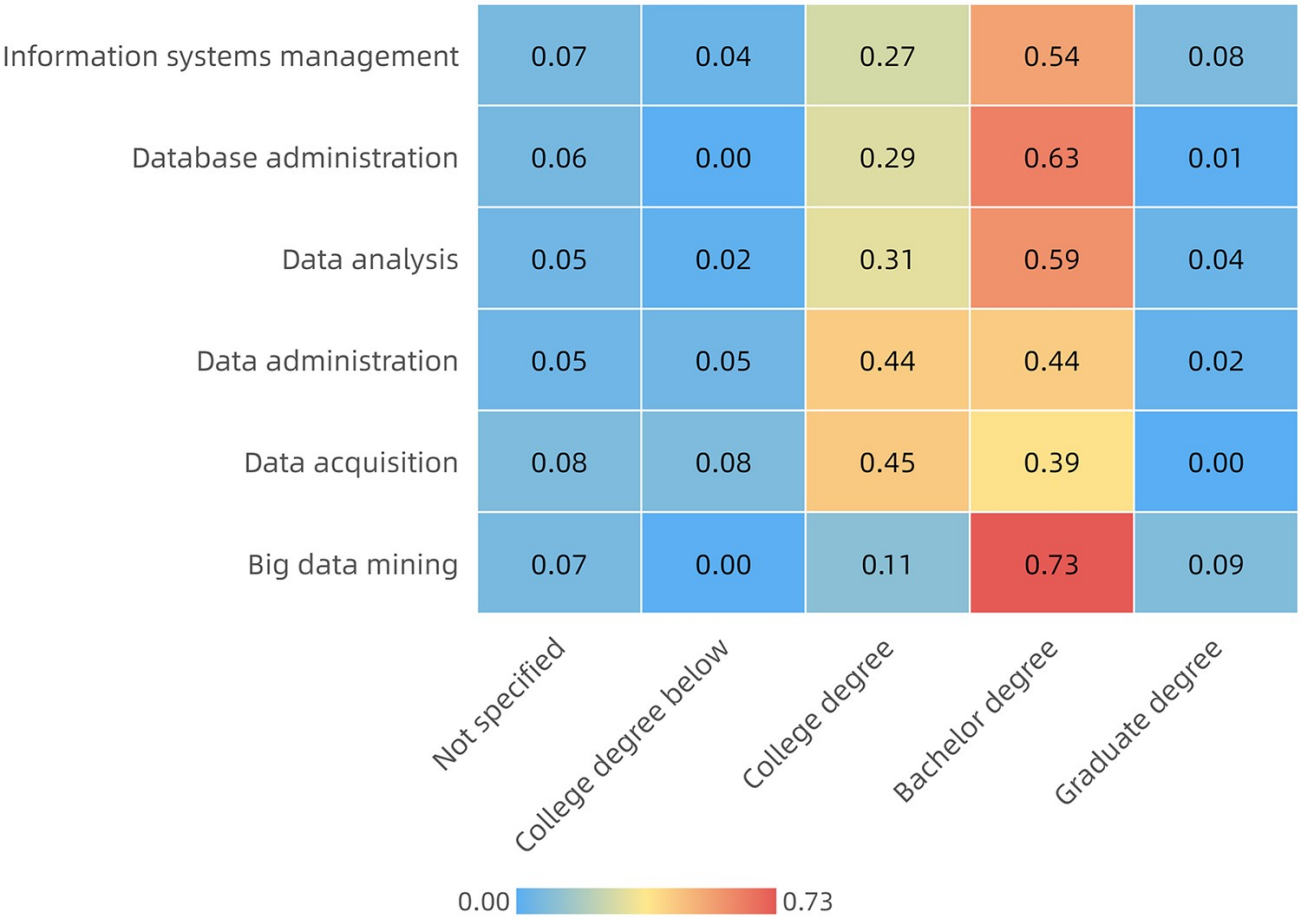
Proportion of educational degrees in each position.

At the same time, the degree of difficulty of the job is also reflected in different requirements for education. As shown in Fig 8, the horizontal axis is the degree of education, the vertical axis is the positions and each grid represents the percentage of the job that requires a degree. It can be briefly summarized that big data mining jobs require 82% of employees to has a bachelor’s or graduate degree. Approximately 60% of the database administrator, data analyst and information systems manager should have a bachelor’s degree or above, and approximately 80% of the data administrators and data collectors should half-and-half have a college degree and bachelor’s degree.

Through co-occurrence analysis of the educational requirements and skill requirements of each position, different skill requirements under different academic qualifications can be counted and ordered. As shown in Table 5, we keep the data analysis method and tool words and remove the nonprofessional skills words and nonspecific analysis method words. In general, the higher the academic requirements, the higher the demand for skills. However, those skill requirements are not very demanding for the corresponding academic qualifications. For example, employees with college degrees and below need to be familiar with offices, especially pivot tables. From the perspective of labor supply, the threshold of engaging DAPET is not high. Studying professional courses and practicing basic skills are the stepping stones of this vocation.

**Table 5:** Requirement of professional skill terms of each educational degree.

Graduate degree	Bachelor degree	College degree and below	Not specified
python	excel	excel	excel
R	sql	office	oracle
machine learning	python	sql	sql
sas	mysql	ppt	office
sql	oracle	oracle	R
office	r	mysql	python
spss	office	word	mysql
logistic regression	ppt	python	ppt
C	Linux	PivotTable	sas
matlab	sas	functions	word
excel	machine learning	backups	C
cluster	word	linux	linux
Java	logistic regression	shell	hadoop

### C. Horizontal analysis

#### What’s the difference between this occupation and other occupations?

**Fig 9:**
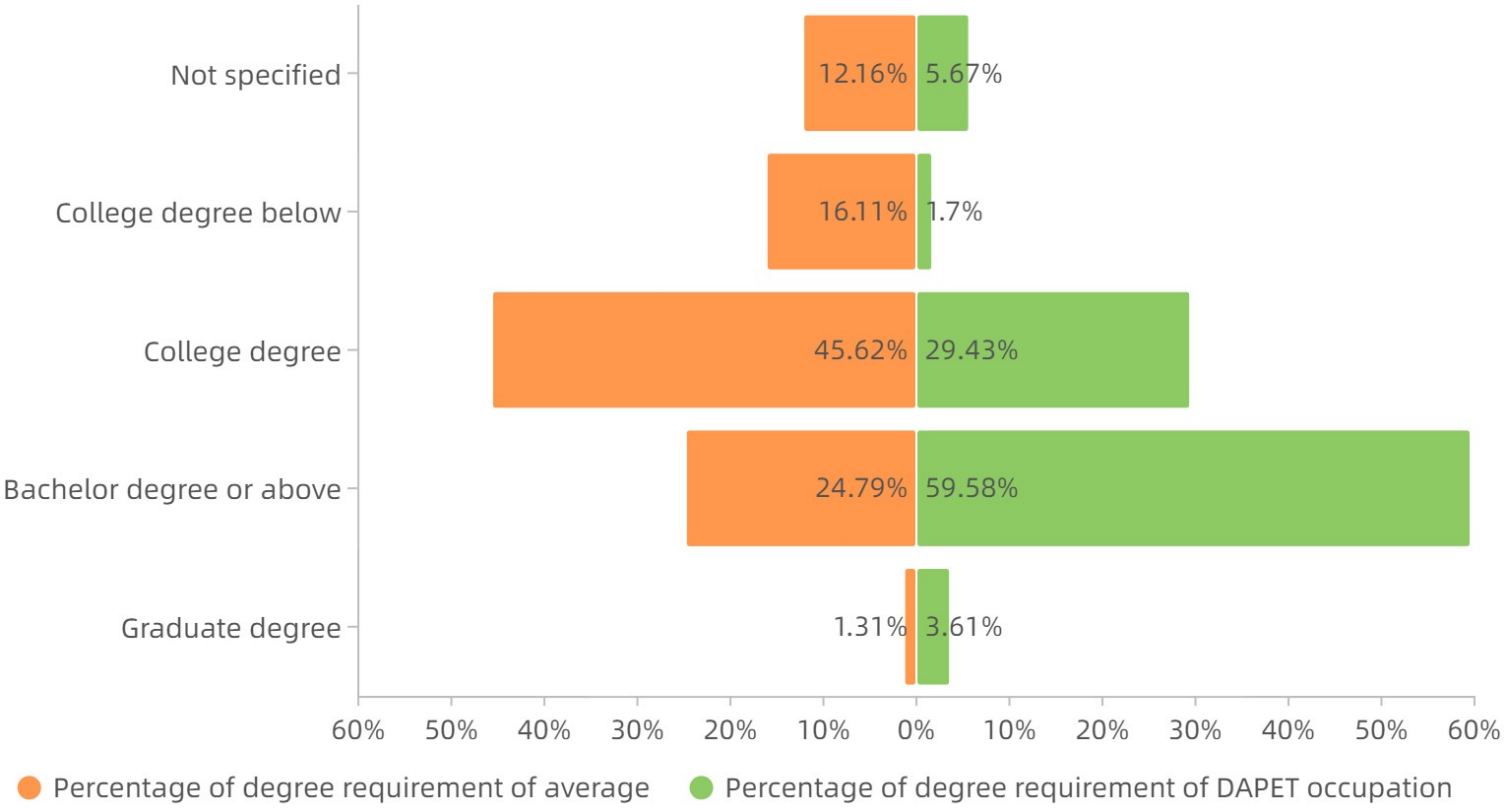
Comparison of distributions of degree requirement.

**Fig 10:**
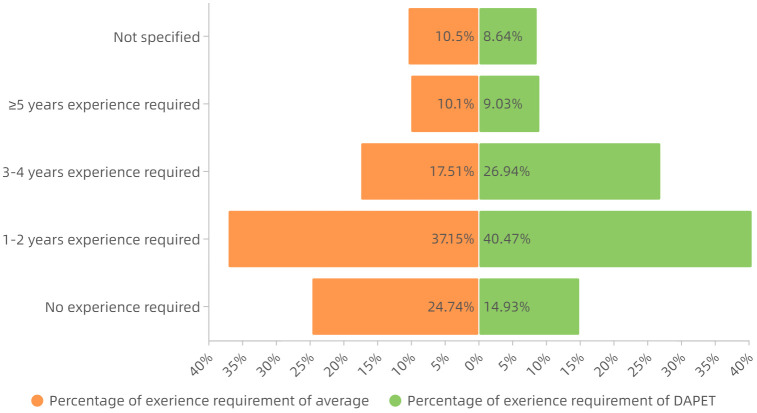
Comparison of distributions of work experience requirement.

1. *Degree requirements analysis*. Statistics on the degree requirements of the DAPET occupation showed that, compared to the average degree demand distribution which was the statistical result of all the data, this occupation had a higher degree requirement. The specific distribution is shown in Fig 9: in the occupational dataset, except for 6% of the job posting data with no educational requirement, 60% and 4% of the data requires bachelor’s degree or graduate’s degree, which is approximately 37 percentage points higher than the average; the proportion of college degree requirements is 29%, 16 percentage points lower than the average; and the proportion requiring a technical secondary degree and high school or below is 2%, 14 percentage points lower than the average. Relatively speaking, this occupation has a higher threshold of employment.
2. *Experience requirements analysis*. According to the statistics of work experience requirements, it was found that compared with the average distribution of work experience requirements which was the statistical result of all the data, the work experience requirements of this occupation were relatively high. The specific distribution is shown in Fig 10: in the occupational dataset, in addition to 9% of the sample data without work experience requirements, only 15% of the job postings data require no prior experience, which is 10 percentage points lower than the average; 40% of the sample data required 1–2 years of experience, 3 percentage points higher than the average; 27% of the sample data required 3–4 years of experience, which is 9 percentage points higher than the average; and 9% of the sample data required 5 or more years of experience, 1 percentage point below the average. In summary, three-quarters of job postings require applicants with 1–4 years of work experience. Thus, students interested in this vocation should also take into account the possible internships of similar jobs, especially for students majoring in statistics, mathematics or computer science.

### D. Occupational profile

In conclusion, we can give a brief profile of the DAPET occupation: technicians engaged in data analysis and processing engineering usually major in Computer Science, Statistics and Mathematics; the positions they engaged are big data mining, data analysis, database administration, information systems management, data administration and data acquisition; core skills they commonly have are communication ability, sense of responsibility, logical thinking, teamwork spirit, responsibility. Professional skills are data analysis ability, data sensitivity, office software, statistical analysis software, programming language and database management software. In particular, the big data mining job requires the mastering of machine learning and high-performance computing technologies. Sixty-four percent of these personnel have a bachelor’s degree or above; three quarters of them have 1–4 years of work experience; their average annual salary is 107,000–168,000 yuan per year; half of the enterprises for which they work are located in Beijing, Shanghai, Guangzhou and Shenzhen.

## Discussion

This paper proposed an occupational portrait based on online recruitment data. To put this into practice, the article took the occupation named data analysis and processing technician numbered GBM2023009 in China’s current occupational classification standard as an example and showed how to mine the micro required information of the labor market about skills, specialties, experience and so on from the data of 51job.com to describe the occupation from multiaspects. The work contributes to the existing studies by (i) proposing a new typology of occupation profile in the occupational information system; (ii) presenting an efficient and automated approach of named entity recognition for information extraction of job advertisements; (iii) providing a decision-making support and guidance for practical management issues such as vocational skills training, educational reform, and social human resource allocation.

Our research has several limitations. First, the limitation stems from the single data source. Although 51job.com which we chose to capture data is a relatively leading website for advertising jobs in China, it may be biased to select only one website. In the future, the study should get information from more websites. The second limitation is the false recruitment information published on the website may affect the authenticity of the experimental results. In China, some companies publish job advertisements to advertise themselves or collect resumes but not recruit. The study did not identify and exclude these bogus data. The third limitation is the biased text of job advertisements. The text of the job advertisement is usually written by human resource managers so that it usually represents a basic skill requirement by organizations but not a specific skills and experience requirement by the occupations.

Several opportunities for future research are opened by this study. This work is preliminary exploratory work to carry out a comprehensive vocational profile. In fact, the approach proposed in this article can be extended to many other occupations. In the future, we will study the classification algorithm to divide the online recruitment data into more occupational data sets according to Chinese occupational classification standards. As the data accumulates each year, we will consider adding time variables to discover the heat changing of the skills over time and analyze more relevance between the occupations and specialties to obtain more information.

## Supporting information

S1 Data (XLS)

S1 File The data analysed. (CSV)
